# On fear of missing out, social networks use disorder tendencies and meaning in life

**DOI:** 10.1186/s40359-023-01342-9

**Published:** 2023-10-26

**Authors:** Christian Montag, Marko Müller, Halley M. Pontes, Jon D. Elhai

**Affiliations:** 1 Department of Molecular Psychology, Institute of Psychology and Education, Ulm University, Helmholtzstr. 8/1, 89081 Ulm, Germany; 2 School of Psychological Sciences, Birkbeck, University of London, London, UK; 3 Department of Psychology and Department of Psychiatry, University of Toledo, Toledo, OH USA

**Keywords:** Social networks use disorder, Problematic social media use, Social media addiction, Well-being, Meaning in life, Fear of missing out

## Abstract

Much research has been conducted on the association between social networks use disorder tendencies (SNUD; i.e. problematic social media use) and well-being. In more detail, a meta-analysis and further research suggest an inverse association between higher SNUD tendencies and lower well-being. In existing studies on social media use and well-being, this association has often been investigated by asking participants about their life satisfaction and/or emotional well-being. A path that has only recently been used to shed further light on SNUD tendencies is the assessment of meaning in life. Against this background, the present study aimed to investigate both the search for and presence of meaning in life in the context of SNUD tendencies in 955 participants. To the best of our knowledge and beyond what has been mentioned, this study is the first to investigate the links between meaning in life variables and state/trait fear of missing out (FoMO), with FoMO being a critical variable known to be linked to the overuse of digital technologies. In a structural equation model, we observed that associations between FoMO and meaning in life variables was mediated by SNUD tendencies. Although the present study is of cross-cultural nature and cannot establish causality, it might be the case that proneness to FoMO might trigger SNUD tendencies and as a consequence, results in more search for meaning in life, while hindering presence of meaning in life.

**Supplementary Information:**

The online version contains supplementary material available at 10.1186/s40359-023-01342-9.

## Introduction

Currently, nearly five billion people use social media services [1]. With the rise of social media and the increasing amount of time spent on social media platforms [2], scientists around the globe try to understand whether social media use impacts mental health and well-being; for a methodological review, see a recent study [3]. Meanwhile, it has been shown that a focus taking into account several person variables might be best to understand whose well-being might suffer from excessive social media use by focusing on factors such as age, gender, personality, and active *vs.* passive use, to name a few variables [4, 5].

Of note, prolonged time spent on social media has shown only weak negative associations with well-being measures [6], underlining that understanding well-being in the context of (excessive) social media use is more complex than such a simple equation. Interestingly, effect sizes of correlations with well-being tend to be slightly larger when problematic use within an addiction framework is used to characterize a person’s excessive use of social media instead of social media use overall [6, 7]. In the present study, we adopt the term Social Networks Use Disorder tendencies (SNUD tendencies) instead of problematic social media use to better align terminologies with the disorder term proposed for Gaming and Gambling Disorder in the ICD-11. As SNUD is not currently officially recognized as a disorder, we examine SNUD tendencies because we investigated a subclinical sample (for further discussion see these studies [8, 9]). Moreover, we stress the relevance of not overpathologizing everyday life behaviors [10].

In general, research investigating the relationship between social media use and well-being is hampered by the jingle jangle fallacy [4] because this relationship can comprise factors such as positive emotions, life satisfaction, or absence of depression and anxiety. In the context of a “positive psychology framework” to understand the consequences of (excessive) social media use, a construct less investigated in the context of SNUD tendencies represents “meaning in life” [11]. Meaning in life can be distinguished into the constructs “searching for meaning life” (Search) and “actually having found meaning in life” (Presence).

Against the background that higher SNUD tendencies are associated with lower well-being, for instance, when assessing well-being with life satisfaction [12], it is relevant to note that searching for meaning in life has been associated with lower well-being, whereas the presence of meaning in life has been associated with higher well-being (in terms of directions of correlations) [13]. Therefore, it is expected that search for meaning in life would be associated with greater SNUD tendencies, while the presence of meaning in life would correlate with lower SNUD tendencies. Recent evidence has shown that the presence of meaning in life is associated with lower SNUD tendencies, but only null correlations were observed with the search for meaning in life [14] (we found these studies after preregistration of the present work). Another study investigated associations between social media use (frequency) in the context of meaning in life but could not find a significant bivariate correlation as presented in Table 1 of their work (but see additional findings with active vs. passive social media use) [15]. That study did not investigate the meaning in life construct as a two-factorial construct (only a focus on presence) and frequency of social media use was explored instead of SNUD tendencies. The present study could also be seen in light of the I-PACE model, which explains the etiology of Internet Use Disorders [16]. I-PACE is an acronym for interaction of person-affect-cognition and execution variables, whereas fear of missing out (FoMO) could be seen either as person-variable, when it would be a trait, but it could also be seen as a cognitive bias resulting in Internet Use Disorder tendencies (here SNUD).

**Table 1:** Means and standard deviations for primary continuous variables separated by gender

Variable	Men (n = 467) M (SD)	Women (n = 488) M (SD)	F(1,953)	p	η^2^_p_
FoMO Trait	12.01 (4.58)	11.74 (4.42)	0.91	0.34	< 0.001
FoMO State	16.51 (7.00)	14.61 (5.71)	21.10	< 0.001	0.02
Presence	24.84 (6.55)	24.42 (6.55)	0.94	0.33	< 0.001
Search	20.21 (8.59)	19.44 (9.09)	1.84	0.18	0.002
SNS-AT	13.58 (6.06)	12.52 (5.07)	8.62	0.003	0.009
Age	45.43 (13.74)	42.66 (14.87)	8.94	0.003	0.009

Note. FoMO = Fear of Missing Out; SNS-AT = Social Networking Sites - Addiction Test. Gender was coded “1” for men, and 2 for women

### Hypothesis of the present study as also mentioned in the preregistration

We expect meaning in life variables to be associated with SNUD tendencies. This is because, it could be that people that are still searching for meaning in life use social media excessively to fill in a void in their lives. For the present study, we model search for meaning in life as the outcome variable, because excessive time spent on social media/SNUD tendencies might hinder finding meaning in life, and the search for meaning in life then needs to continue. On the other hand, the presence of meaning in life could also be seen as a buffer against developing SNUD tendencies, or it is possible that the absence of SNUD tendencies might pave the way for the presence of meaning in life. The latter idea is supported by empirical evidence showing that reducing social media/smartphone use can elevate well-being/reduce negative emotionality [17, 18]. The work by Hunt et al. [18] also explicitly mentions FoMO as a relevant psychological mechanism, which might be key to understanding the relationship between social media use and well-being (and other studies showed that FoMO is robustly associated with higher problematic SNUD/smartphone use disorder tendencies [19, 20, 21]. Of note, FoMO can be seen as the fear that others have a rewarding time from which one is absent [20]. Therefore, we also assessed FoMO in the present study and expected it to be positively associated with the search for meaning in life and SNUD tendencies. Further negative associations between FoMO and the presence of meaning in life are expected. The mentioned hypotheses have also been preregistered before the data collection via the Open Science Framework (see Methods for the direct link).

## Methods

### Participants

1151 participants were recruited for the present study via the research institute Bilendi GmbH. 467 males and 488 females were included in the final sample (see data cleaning steps below). The participants were recruited with the aim to be balanced for gender (about 50% males and 50% females) with a large age range, starting with 18 years. The data collection was done for several different research projects, whereas here the focus is on FoMO, SNUD tendencies and meaning in life. The study had approval from the ethics committee at Ulm University (Ulm, Germany) and the present research with its questions was preregistered at the Open Science Framework: https://osf.io/bqdeu - the data can be downloaded here: https://osf.io/fg62k/.

### Questionnaires

Several questionnaires were administered in this study. SNUD tendencies were assessed using the German version of the Social Networking Sites - Addiction Test (SNS-AT; please note that we were imprecise in the preregistration as we mentioned a different, related questionnaire there). The scale consists of six items rated on a five-point Likert scale ranging from 1 = strongly disagree to 5 = strongly agree (see also the wording of the German translation in the supplement). The scale was developed based on the six components model of addiction that include salience, tolerance, mood modification, relapse, withdrawal and conflict in line with the Bergen Facebook Addiction Scale (BFAS) [22], whereby in the original scale only Facebook use disorder tendencies were assessed (please note that a follow-up study already used the social media wording: Bergen Social Media Addiction Scale (BSMAS); [23]). In contrast to the BSMAS, the SNS-AT evaluates SNUD tendencies via first-person statements from the “I”-perspective in addition to improving the overlap of the items with the components model of addiction developed by Griffiths [24]. Internal consistency in our sample are excellent: α = 0.88 and ω = . 92. All items used can be found in the supplement.

Further, participants filled in the German version of the FoMO scale [25], which consists of 12 items with a five-point answer format ranging from 1 = strongly disagree to 5 = strongly agree. Five items assess a general trait FoMO score, and seven items assess a state FoMO score, which is related to FoMO in the digital realm. Higher scores indicate greater trait or state FoMO. Internal consistency is as follows: α = 0.82 and ω = 0.91 for trait FoMO, and α = 0.89 and ω = 0.92 for state FoMO. Finally, all participants answered the meaning in life questionnaire [11] consisting of 10 items being answered on a seven point answer scale ranging from 1 = do not agree at all to 7 = completely agree. The German version was taken from Steger’s website (http://www.michaelfsteger.com/?page_id=13) where an overview on different languages to use is provided. Five items assess the concept of the Presence of Meaning in Life (with one reverse-coded item) and five items Search for Meaning in life. Higher scores indicate either a higher presence or higher search for meaning in life. Internal consistency is as follows: α = 0.90 and ω = 0.92 for presence of meaning in life (Presence), and α = 0.96 and ω = 0.97 for search for meaning in life (Search).

### Data cleaning

From a dataset of n = 1151, we first excluded participants who stopped and then restarted the survey later, resulting in 1097 participants. Additionally, only participants who endorsed using social media were included in the study. This reduced the sample size to 1005. Furthermore, participants belonging to a third or diverse gender represented only a very small group and were also excluded because no robust statistics could be calculated with this small value of n, resulting in 1001 participants. We next excluded participants reporting inadequate German language proficiency (resulting in 998 participants), for not indicating consent in a dedicated item we used (resulting in 997 participants), or failing an attention/conscientiousness check (resulting in 990 participants). Finally, participants with the same consecutive answers to at least 15 items were also excluded (resulting in a final sample n = 955).

### Data analysis

We used R software 4.3.0 for data cleaning and descriptive and correlational analyses. We used several R packages, including *pastecs* (descriptives), *careless* (inattentive responding), *mice* (for missing item-level imputation), *fmsb* and *GPArotation* (internal consistency), *corrplot* (correlations), and *sjstats* (ANOVA effects). We used *mice* to impute missing item-level data for each scale (1–2 missing items were found per scale). We summed scale scores for descriptive and correlational analyses. We computed a Pearson correlation matrix for scale scores and age, and tested for gender differences using ANOVAs.

We used Mplus 8.9 for Confirmatory Factor Analyses (CFA) for the psychological scales. For the meaning in life two-factor Search/Presence CFA, we treated items as continuous, using a Pearson covariance matrix, linear regression-based factor loadings, and maximum likelihood estimation with robust standard errors. For the SNS-AT single-factor CFA, and FoMO two-factor trait/state CFA, we treated items as ordinal (because of their 5-point scale), using a polychoric covariance matrix, probit factor loadings, and weighted least squares estimation with a mean- and variance-adjusted chi-square. We correlated the residual error variances for FoMO items 1 and 2 which are very similar in content (fearing that others vs. friends are having more rewarding experiences). We judged model fit based on established benchmarks, including the Comparative Fit Index (CFI) and Tucker-Lewis Index (TLI) > 0.94, Root Square Error of Approximation (RMSEA) < 0.06, and Standardized Root Mean Square Residual (SRMR) < 0.08 [26].

**Fig. 1:**
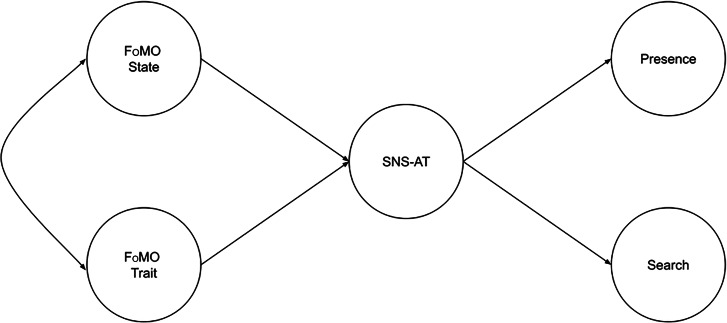
Hypothesized model. (Note. Circles denote latent variables. Factor loading paths/arrows from the latent variables to their respective observed variables are not displayed, to avoid clutter (the data can be found via the OSF data link provided in the method section). FoMO = Fear of Missing Out; SNS-AT = Social Networking Sites - Addiction Test

We next tested the full structural equation model (SEM) shown in Fig. 1, using the estimation approach above. We then tested mediation effects by computing cross-products of path coefficients, implementing the Delta method and 1000 non-parametric bootstrapped replications for indirect effect standard error estimation.

## Results

**Fig. 2:**
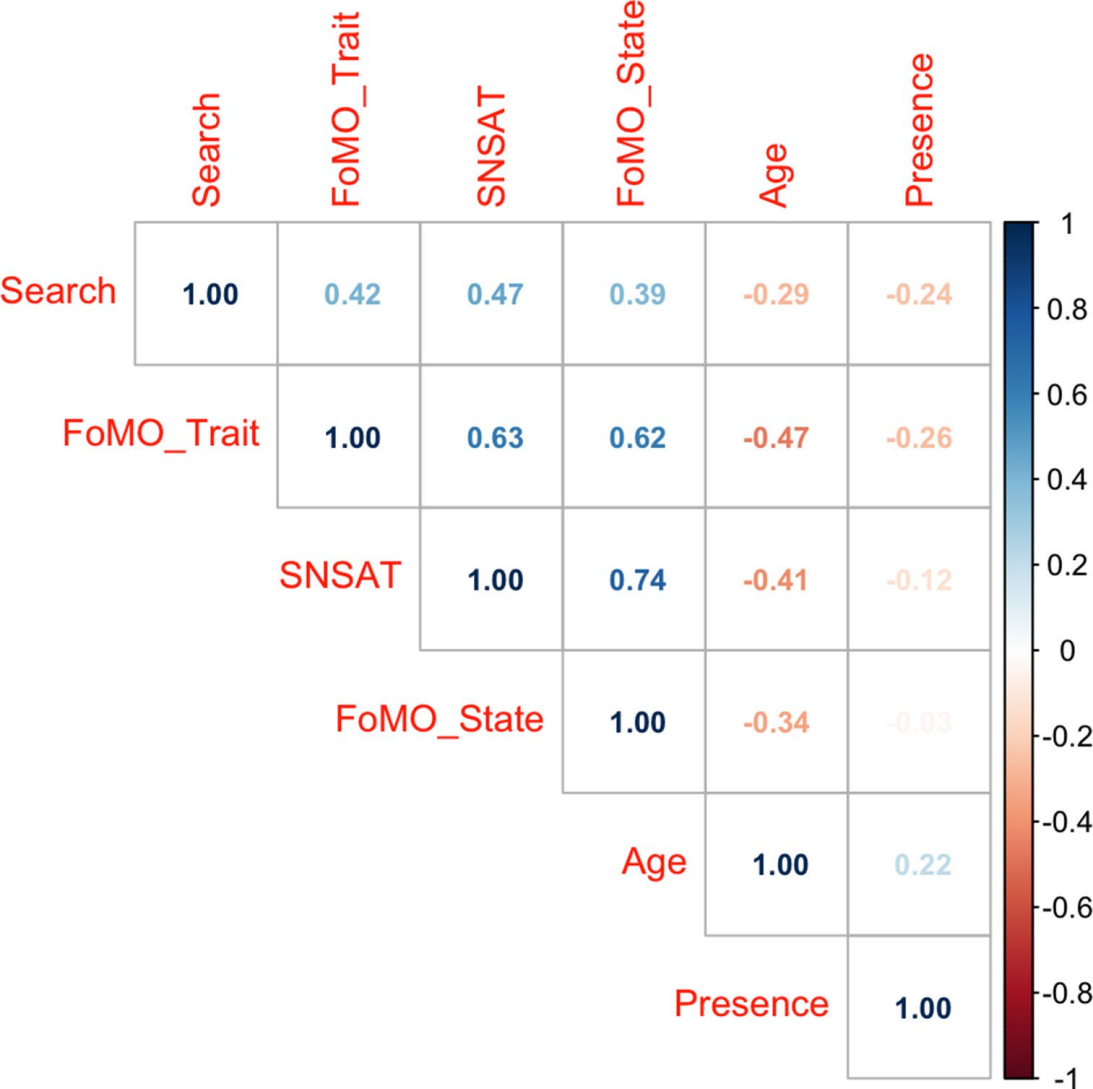
Pearson correlations between variables. (Note. FoMO = Fear of Missing Out; SNS-AT = Social Networking Sites - Addiction Test. The figure is a correlation heatmap, with correlations displayed, with darker colors indicating stronger relationships (blue for positive, and red for inverse relations). All correlations were statistically significant (p < .001) except between Presence and state FoMO (p = .32)

Descriptive statistics for scale scores and age are displayed in Table 1, along with between-group differences on gender (which were all small in magnitude). Figure 2 displays the correlation matrix heatmap. Variables were correlated in the expected direction, with Search positively and Presence negatively related to the other scales (not considering age).

The SNS-AT single-factor CFA fitted well, except for RMSEA as expected with ordinal data [27], robust χ^2^(9) = 256.30, p < .001, CFI = 0.97, TLI = 0.95, SRMR = 0.03, RMSEA = 0.17 (95% CI: 0.15-0.19). The two-factor FoMO CFA fitted well, robust χ^2^(52) = 502.44, p < .001, CFI = 0.98, TLI = 0.98, SRMR = 0.04, RMSEA = 0.10 (95% CI: 0.09-0.11). Finally, the two-factor meaning in life CFA also showed adequate fit, χ^2^(34) = 141.58, p < .001, CFI = 0.98, TLI = 0.97, SRMR = 0.04, RMSEA = 0.06 (95% CI: 0.05-0.07).

**Fig. 3:**
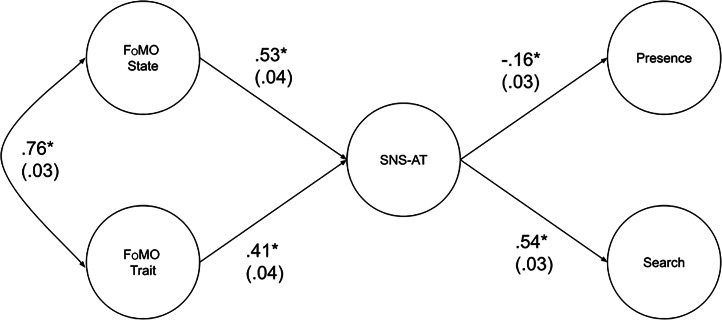
SEM model with standardized path coefficients. Note. Circles denote latent variables. Factor loading paths/arrows from the latent variables to their respective observed variables (and their coefficients) are not displayed, to avoid clutter (the data can be found via the OSF data link provided in the method section). Numbers in parentheses are standard errors. FoMO = Fear of Missing Out; SNS-AT = Social Networking Sites - Addiction Test. * p < .001

The full SEM fit reasonably well, robust χ^2^(343) = 2004.08, p < .001, CFI = 0.94, TLI = 0.93, SRMR = 0.06, RMSEA = 0.07 (95% CI: 0.07-0.07). Figure 3 displays the SEM model results with standardized path coefficients. We tested a model alteration to Fig. 1, by adding paths from state FoMO and trait FoMO directly to Presence and Search. This model alteration fit significantly better, robust χ^2^_diff_(4) = 131.41, p < .001, but the magnitude of difference was small based on previous benchmarks, CFI_diff_ = 0.01 [28].

In mediation testing based on the original model, we found that the SNS-AT significantly mediated relations between trait FoMO and both Search (β = 0.22, SE = 0.03, p < .001) and Presence (β = − 0.07, SE = 0.02, p = .001). The SNS-AT also mediated relations between state FoMO and both Search (β = 0.29, SE = 0.03, p < .001) and Presence (β = − 0.09, SE = 0.02, p < .001).

See the supplemental text attached to this submission for a version of the model in our original preregistration.

## Discussion

The present preregistered study aimed to understand the relationships between FoMO, SNUD tendencies, and meaning in life (Presence/Search). In line with the preregistration, we observed positive associations between FoMO, SNUD, and search for meaning in life. In the realm of FoMO we investigated both trait and state facets as proposed in a recent study [25]. Interestingly, the trait FoMO facets being linked to FoMO in general (and not with a focus on the online realm) showed lower associations with SNUD tendencies compared to the state (online) FoMO variable. This makes sense because these SNUD tendencies are linked to the online context. In contrast, it seems that trait FoMO is of more relevance than state FoMO to understand individual differences when investigating search for/presence of meaning in life. This is also understandable, because a more general FoMO factor should also be linked to a broader life variable such as search for meaning in life. Further, in line with the preregistration, we observed negative associations between presence of meaning in life and SNUD / FoMO tendencies. In so far having found meaning in life is clearly positive in terms of lower FoMO related negative affect and lower SNUD tendencies. Interestingly, the SEM findings revealed that our model assumption with meaning in life variables being the outcome variables, the FoMO variables being the predictors and SNUD tendencies being the mediator turned out to be valid (this fits well with the I-PACE model, [16]). Hence, higher trait/state FoMO was associated with higher search for meaning in life and lower presence of meaning in life, with SNUD tendencies being a mediator.

Since the study conducted was cross-sectional, it is not possible to disentangle cause and effect between the mentioned variables. Nevertheless, we proposed in our preregistration to also investigate an alternative mediation model with meaning in life variables being the outcome variables, SNUD tendencies being the predictor and trait/state FoMO the mediator variables. Such a model would make sense, if one imagines that persons with higher SNUD tendencies spend excessive time on social media platforms, whereas the platform design triggers FoMO [29]. For instance, social media platforms try to lure in users via push notifications, further exacerbating FoMO on their services. As a consequence of both SNUD tendencies and negative FoMO affect, persons would then have difficulties to find meaning in life (Presence), and the search for such a meaning in life has to continue. Such mediation effects could also be observed and are presented in the supplementary material. Aside from this, we also deem it plausible that those with higher FoMO tendencies have higher search for meaning in life activities (FoMO fueling search for meaning in life) and less presence of meaning in life. Such associations could then be mediated by SNUD tendencies. Several studies have suggested that FoMO is a risk factor for Internet Use Disorder tendencies [19, 20], including SNUD [21] and smartphone use disorder tendencies. The present study supports these ideas. In general, the FoMO process needs to be seen a critical element in research dealing with SNUD, because FoMO seen as a personality trait can make people more vulnerable to overuse social media [20]. On the other hand, the industry uses the FoMO mechanism to bring people to their platforms. Hence, it is of importance to think in the future about healthy platform design and interventions on how to reduce FoMO. This could fall in a larger context of re-vamping social media in more healthy ways [30].

This study adds to the limited available literature investigating meaning in life in the context of (excessive) social media use and adds relevant data to the discussions around well-being in the online realm [4]. Our findings support the observation that higher SNUD tendencies are linked to a lower presence of meaning in life [14], but please note that this study did not find an association with the search for meaning in life.^1^ In contrast, we observed such an inverse association but the effect sizes were larger between SNUD and search for meaning in life compared to SNUD and presence for meaning in life (but going in contrary directions). Finally, we also mention new work that investigated problematic social media use (with a focus on Facebook and Instagram) in the context of meaning in life. As they used an extended questionnaire providing insights into concepts such as meaning anxiety and confusion, the study findings cannot be directly compared to the present work [31]. The same is in parts true for another study by Helm et al. [15], as they only investigated the presence of meaning in life variable.

This study has several limitations. First, it is a study based on self-reporting methodology, which goes along with the usual possibilities of biases, including answering in a socially desirable fashion. Second, this study is limited by its cross-sectional nature; hence, we cannot disentangle the effects of the assessed variables. Third, it would be interesting to add digital phenotyping and mobile sensing principles [32] to the present study to obtain insights into objective social media use parameters.

In conclusion, the present study is one of the first to investigate SNUD tendencies in the context of meaning in life. Further, to our knowledge, this is the first study to investigate the important relationship between SNUD tendencies and meaning in life with trait and state FoMO. One of the most relevant findings encountered in the present study is the observation that the association between higher FoMO variables and lower presence of meaning in life is mediated by SNUD tendencies. As it is well described that the industry also uses the FoMO mechanism to lure people in to their social media platforms, [29] the present study findings could also be seen in this light, that it is important to design healthier social media platforms that do not exploit the FoMO mechanism [33, 34].

### Electronic supplementary material

Below is the link to the electronic supplementary material.

Supplementary Material 1

## Data Availability

The data is available at the Open Science Framework: https://osf.io/fg62k/.

## Footnotes

1 We found these studies after preregistration of the present work.
