# Correction: On fear of missing out, social networks use disorder tendencies and meaning in life

**DOI:** 10.1186/s40359-023-01428-4

**Published:** 2023-11-08

**Authors:** Christian Montag, Marko Müller, Halley M. Pontes, Jon D. Elhai

**Affiliations:** 1 Department of Molecular Psychology, Institute of Psychology and Education, Ulm University, Helmholtzstr. 8/1, 89081 Ulm, Germany; 2 School of Psychological Sciences, Birkbeck, University of London, London, UK; 3 Department of Psychology and Department of Psychiatry, University of Toledo, Toledo, OH USA

**BMC Psychology (2023) 11:358**

10.1186/s40359-023-01342-9

Following publication of the original article, the authors flagged that in the Questionnaires subsection of the Methods the ‘phi’ symbol had erroneously been used instead of the ‘omega’ symbol in the following (now correct) instances:

α=0.88 and ω=.92.

α=0.82 and ω=0.91 for trait FoMO, and α=0.89 and ω=0.92 for state FoMO

α=0.90 and ω=0.92 for presence of meaning in life (Presence), and α=0.96 and ω=0.97 for search for meaning in life (Search).

The published article has been corrected. The authors thank you for reading this erratum and apologize for any inconvenience caused.

