# Supplementary material for: On fear of missing out, social networks use disorder tendencies and meaning in life
Source: BMC Psychol. 2023 Oct 26;11:358. doi: 10.1186/s40359-023-01342-9 (PMC10601113; doi:10.1186/s40359-023-01342-9)
Supplement: Supplementary file 1 — Supplementary Material 1 [file 40359_2023_1342_MOESM1_ESM.docx]

**Supplementary Material**

We also tested a model variation where we switch the sequence of the FoMO factors and the SNS-AT. This full structural equation model fit reasonably well, robust χ^2^(342) = 1711.732, p < .001, CFI = .95, TLI = .94, SRMR = .05, RMSEA = .07 (95% CI: .06-.07). Supplemental Figure S1 displays the SEM model results with standardized path coefficients.

In mediation testing based on the original model, we found that the FoMO State significantly mediated relations between the SNS-AT and both Search (β = .18, SE = .06, p = .002) and Presence (β = .36, SE = .06, p < .001). FoMO Trait also mediated relations between the SNS-AT and both Search (β = .30, SE = .06, p < .001) and Presence (β = -.50, SE = .05, p < .001).

Supplemental Figure S1. SEM model with standardized path coefficients.

**

**

Note. Circles denote latent variables. Factor loading paths are not displayed, to avoid clutter (but can be found via the OSF data link in the method section). Numbers in parentheses are standard errors. FoMO=Fear of missing Out; SNS-AT: Social Networking-Site-Addiction-Test.

* p < .001

Supplemental Table 1: Social Networking Sites - Addiction Test (SNS-AT): English Version

***Instructions***: The following statements describe peoples’ experiences when using online social networking sites such as *Instagram,* *Snapchat, Twitter, Facebook, TikTok, Youtube* and the like. Please read all the statements below and choose the response that best describes your experience using online social networking sites in the past 12 months.

|  | **Strongly Disagree** | **Disagree** | **Neither Agree or Disagree** | **Agree** | **Strongly Agree** |
| --- | --- | --- | --- | --- | --- |
| 1. I have spent too much time thinking about using social networking sites. | 1 | 2 | 3 | 4 | 5 |
| 2. I have increased my social networking sites usage to feel satisfied. | 1 | 2 | 3 | 4 | 5 |
| 3. I have used social networking sites to help me feel better when I was low. | 1 | 2 | 3 | 4 | 5 |
| 4. I have tried to stop using social networking sites several times but was not able. | 1 | 2 | 3 | 4 | 5 |
| 5. I have experienced unpleasant feelings when unable to use social networking sites. | 1 | 2 | 3 | 4 | 5 |
| 6. I have experienced many problems in my life because of my social networking sites usage. | 1 | 2 | 3 | 4 | 5 |
| Theoretical Domains: 1 = Salience; 2 = Tolerance; 3 = Mood Modification; 4 = Relapse; 5 = Withdrawal; 6 = Conflict. | | | | | |

Supplemental Table 2: Social Networking Sites - Addiction Test (SNS-AT): German version (but with a focus on the term social media)

Please note that the instruction differs a bit compared to the English version above. The version depicted below was applied in the present work. Of note, in the German wording we speak of social media.

Instruktion: Im Folgenden sind Aussagen im Zusammenhang mit der Nutzung von sozialen Medien wie *Instagram,* *Snapchat, Twitter, Facebook, TikTok, Youtube* und ähnlichen aufgeführt. Bitte lesen Sie die Aussagen sorgfältig und wählen Sie die Antwort, die Ihre Erfahrungen mit sozialen Medien in den letzten 12 Monaten am besten beschreibt.

|  | **Stimme überhaupt nicht zu** | **Stimme nicht zu** | **weder**  **noch** | **stimme zu** | **Stimme voll und ganz zu** |
| --- | --- | --- | --- | --- | --- |
| 1. Ich habe zu viel Zeit mit Nachdenken über soziale Medien verbracht. | 1 | 2 | 3 | 4 | 5 |
| 2. Ich habe die Nutzung sozialer Medien erhöht, um mich zufriedener zu fühlen. | 1 | 2 | 3 | 4 | 5 |
| 3. Ich habe soziale Medien dafür genutzt, mich besser zu fühlen, wenn es mir schlecht ging. | 1 | 2 | 3 | 4 | 5 |
| 4. Ich habe einige Male versucht soziale Medien nicht mehr zu nutzen, es aber nicht geschafft. | 1 | 2 | 3 | 4 | 5 |
| 5. Ich hatte schlechte Gefühle, wenn ich keine sozialen Medien nutzen konnte. | 1 | 2 | 3 | 4 | 5 |
| 6. Ich habe in meinem Leben durch die Nutzung sozialer Medien viele Probleme bekommen. | 1 | 2 | 3 | 4 | 5 |
| Theoretische Domänen: 1 = Salienz; 2 = Toleranz; 3 = Stimmungs-Modifikation; 4 = Rückfall; 5 = Entzugserscheinungen; 6 = Konflikt. | | | | | |
